# Use of Telemedicine in Addiction Treatment: Current Practices and Organizational Implementation Characteristics

**DOI:** 10.1155/2018/3932643

**Published:** 2018-03-11

**Authors:** Todd Molfenter, Roger Brown, Andrew O'Neill, Ed Kopetsky, Alexander Toy

**Affiliations:** ^1^Department of Industrial and Systems Engineering, University of Wisconsin-Madison, 1513 University Avenue, Madison, WI 53706, USA; ^2^Schools of Nursing, Medicine and Public Health, University of Wisconsin-Madison, 4187 Cooper Hall, 701 Highland Avenue, Madison, WI 53705, USA; ^3^Stanford Children's Hospital, 725 Welch Road, Palo Alto, CA 94304, USA

## Abstract

Telemedicine applications offer innovative approaches for treating and reducing the effects of substance use disorders (SUDs). This analysis assessed the interest in and use of 11 telemedicine applications in a sample of 363 SUD organizations in the United States of America. Fifty percent of the organizations expressed high rates of interest in seven of the telemedicine applications, demonstrating the appeal of telemedicine within this field. The top three self-reported telemedicine applications being used were (1) computerized screening/assessments (44.6%), (2) telephone-based recovery supports (29.5%), and (3) telephone-based therapy (28.37%). The greatest gaps between interest and use were for (1) texting appointment reminders (55.2% differential), (2) mobile apps for posttreatment recovery (46.6% differential), and (3) recovery support chats (46.6% differential). A Latent Class Analysis (LCA) of the organizations' telemedicine use behavior identified three groupings: “Innovators” that were using a range of technologies (*n* = 27, 7.4%); “Technology Traditionalists” that limited their use to telephone, video, and web portal technologies (*n* = 101, 27.8%); and “Low Tech” that had low overall technology use (*n* = 235, 64.7%). Future studies should build on how telemedicine could be applied in SUD settings, organizational behaviors towards its adoption, and telemedicine's effect on treatment adherence and clinical outcomes.

## 1. Introduction

Telemedicine technologies could alter substance use disorder (SUD) treatment service delivery patterns and outcomes. Texting [1], videoconferencing [2], mobile apps [3], web-based treatment supports [4], telephone-based support [5], and use of virtual worlds [6] have all been applied in SUD treatment settings. Web-based computer and mobile device-delivered treatment supports have demonstrated superior outcomes to traditional care [7–9], while treatment delivered through videoconferencing has resulted in similar treatment outcomes and patient satisfaction when compared to traditional face-to-face care [10]. Telemedicine services increase access to service and can provide valuable support when patients are outside of the therapeutic setting and making decisions to use or not use addictive substances. Despite increased use of technology within society at large, adoption of video and mobile telemedicine technologies in SUD care was found to be <1% in a 2012 analysis [11]. Blumenthal & Tavenner (2010) [12] found that electronic health record adoption rates were lowest in SUD services. Molfenter et al. (2015) described the technologies being applied in SUD settings. Yet the level of interest in and use rates of these technologies in SUD settings are not known. Barriers and facilitators to adopting telemedicine in SUD treatment need to be better understood.

Existing technology adoption research has discovered that many factors can affect decisions to adopt and continue to use a technology. At the individual level, the Technology Acceptance Model describes how user acceptance affects patients' and clinicians' willingness to abandon traditional practices in favor of new technologies [13–15]. Beyond the individual level, explanatory models of organizational decisions to adopt a technology have emerged based on two prominent frameworks: diffusion of innovations and the technology-organization-environment framework [16]. These models describe the fundamental role of management support and how factors such as clinical workflow, regulatory policy prohibiting and facilitating use, concerns regarding information security, and financial/reimbursement policy towards the technology affect the decision to purchase, implement, and use a technology [17, 18]. The role of these factors in an SUD treatment organization's technology adoption and the comparative impact of these factors in any healthcare setting have received limited investigation.

### 1.1. Study Aims

The aims of this study are as follows:

- Assess level of interest in using certain telemedicine applications in SUD treatment settings.
- Assess use rates of telemedicine applications in SUD treatment settings.
- Determine if organizational technology adoption behavior profile groupings can be developed, based on an organization's technology use characteristics.
- Assess what organizational factors influence an organization's technology adoption behavior.

The specific aims seek to build upon the current scientific base by (a) expanding beyond current technology adoption research in SUD services that primarily focuses on the efficacy of specific technology applications but does not explore adoption behaviors, (b) building upon the diffusion of innovations literature, which addresses organizational factors associated with adoption and classifies organizations based how quickly they adopt innovations (innovators, early adopters, early majority, etc.), to determine if organizational clusters of technology adoption behaviors emerge based on the types and number of technologies adopted, and (c) adding to the technology adoption literature by confirming or denying how organizational factors such as leadership style, staff cohesion, interdepartment cooperation, organizational history of innovation, and level of internal turbulence influence technology adoption.

## 2. Materials and Methods

*Design*. The study implemented a cross-sectional survey of telemedicine technology interest, use, and organizational readiness for technology adoption traits in SUD organizations in the United States. The study was approved by the University of Wisconsin's Health Sciences Institutional Review Board.

*Procedure and Participants*. Eight states participated in the study: Illinois, Iowa, Kentucky, Massachusetts, Ohio, Oklahoma, Oregon, and South Carolina. Between 10/1/15 and 1/30/16, each of the eight states' addiction treatment authorities e-mailed an invitation to participate, with the on-line survey link, to the CEOs or chief clinical officers of 551 SUD treatment organizations. Survey results were tabulated by the University of Wisconsin-Madison, and respondents were informed that all results would be kept confidential, with the states only receiving aggregate data feedback. A total of 363 public SUD treatment organizations from 8 states completed the survey (Table 1). Surveys were sent to providers that received grant funds from the Substance Abuse Prevention and Treatment Block Grant (SABG) and had greater than 100 admissions per annum. The SABG block grant is a federally supported block grant that is intended to serve the underserved in the United States [19]. The overall return rate was 65.5%, with rates of return ranging from 42.4% in Oregon to 100% in Kentucky, Iowa, and South Carolina.

*Data Measures*. Participants completed an inventory that assessed their interest in and use of 11 telemedicine technologies, based on Molfenter et al. (2015) and technologies that state participants requested. The technologies assessed were (a) computerized screening and assessment tools, (b) texting appointment reminders, (c) texting motivational messages, (d) organizational web portal for patients to use, (e) video-based therapy, (f) mobile apps for use during treatment, (g) mobile apps for posttreatment recovery, (h) secure recovery support chats, (i) telephone-based therapy, (j) telephone-based posttreatment recovery supports, and (k) virtual worlds for treatment. The survey asked what technologies the organization was currently using (e.g., do you currently use this technology? (yes/no)) and what technologies they were interested in using (e.g., what is your interest in using this technology?) (using a Likert Scale with 1 = very low and 5 = very high). The organizational technology implementation characteristics were assessed using organizational-based parameters from the Readiness for Implementation Scale (RIS) (with a Likert Scale of 1 = strongly disagree and 5 = strongly agree). The RIS has prospectively and retrospectively predicted the implementation of e-health systems [20]. The RIS elements selected addressed organizational variables found to align with creating an environment receptive to technology implementation: leadership style, staff cohesion, interdepartment cooperation, organizational history of innovation, and level of internal turbulence [21]. Lastly, a set of technology adoption concerns in SUD settings discovered by Molfenter et al. (2015) [6] was also assessed. This section of the assessment measured patient and counselor attitudes towards the technology, regulatory barriers to implementing the technology, and the technology's impact on workflow, information security, reimbursement, and regulatory policy (with a Likert Scale of 1 = strongly disagree and 5 = strongly agree).

*Data Analysis*. Frequency counts, reported in percentages, were used to report technology use and interest. Means were used to report assessed organizational readiness characteristics and technology implementation concerns. A Latent Class Analysis (LCA) was conducted to profile groupings of SUD treatment organizations based on organizations' use of the technologies listed in Table 2. LCA is widely used to detect homogeneity in a potentially heterogeneous group through evaluating and then minimizing associations among responses across a set of ordered categorical indicators. This determines if organizational groupings of technology use exist based on their self-reported use patterns. We used Mplus version 7.11 (L. K. Muthen and B. O. Muthen, 1998–2012) [22] and applied the basic LC cluster model of

(1) $$\begin{array}{c} f\left(\;y_{i}\;|\;\theta \;\right)=\overset{K}{\underset{k=1}{\sum }}\pi_{k}\overset{J}{\underset{j=1}{\prod }}f_{k}\left(\;y_{ij}\;|\;\theta_{jk}\;\right), \end{array}$$

where *y*_*i*_ denotes an object's scores on a set of observed variables, *K* is the number of classes, and *π*_*k*_ denotes the prior probability of belonging to latent class *K* or, equivalently, the size of class *K*, where *J* denotes the total number of indicators and *j* a particular indicator. To determine the number of classes, we used various information criteria such as Akaike Information Criteria (AIC), sample size adjusted AIC, Bayesian Information Criteria (BIC), and Consistent AIC (CAIC) (see Rissanen, 1978 [23]; Sclove, 1987 [24]; Fraley and Raftery 1998 [25]). The smaller the BIC, AIC, adjusted AIC, and CAIC, the better the model fit.

The number of classes was chosen based on the following selection criteria: (1) interpretability; (2) parsimony; (3) lowest information criteria scores (AIC, adjusted AIC, BIC, and CAIC); (4) entropy > 0.7; (5) average posterior probability in each class >0.75 and no more than 10% overlap/cross-membership between noncontiguous classes; and (6) at least 2.5% of the total sample size in each class using parameters from Nylund et al. (2007) [26] and Collins and Lanza (2013) [27].

After latent class groupings were identified, we were interested in using these latent class variables for a further analysis to explore the possible impact of the different identified latent class groupings and the study's measures assessing organizational readiness and technology implementation concerns. For this auxiliary analysis, we used the Bolck, Croon, and Hagenaars (BCH) method [28, 29] to explore differences between selected study measures and each of the identified individual classes discovered in the LCA.

## 3. Results

Among the 11 technologies assessed from the 363 organizations, the average percentage of organizations that had high interest (or high or very high interest on the Likert Scale) in the different technologies ranged from 35.54% for virtual worlds to 69.97% for computerized screening/assessments. The overall average high interest in all the technologies listed in Table 2 was 37.10%. The percentage use of the different technologies was less than the percentage of high interest in their use. Virtual worlds had a higher percentage of high interest (35.54%), but a low percentage of actual use (.55%). Computerized screening assessments had a high percentage of high interest (69.7%) with an actual use of 44.63%, the highest among the technologies assessed. On average, the difference between the percentage of organizations that had high interest and actual use was 37.32%, with over a third of the organizations having high interest in a technology, but not using it. Texting appointment reminders had the largest gap (55.18%) between high interest and actual use, while the lowest gap between high interest and actual use was in telephone-based therapy (20.67%).

Assessed responses tended to be rated high for several of the organizational technology implementation characteristics. The following technology implementation traits ranked greater than 4 on a 5-point Likert Scale, with 5 being strongly agree: (a) our clinicians and support staff work well together (4.23); (b) our departments work cooperatively together (4.19); (c) our leaders are innovative (4.11). The following traits had lower scores on the Likert Scale: (a) our organization has a history of successful innovation (3.89) and (b) there is a high degree of turbulence in our organization (2.37; reverse coded).

The results from the technology implementation concerns assessment ranged from 2.62 to 3.90 on the 5-point Likert Scale, with 5 being critical, 4 being very high, and 3 being a high concern (Table 3). Information security was the highest concern, with a 3.9 score (representing “very high” concern), and patient attitude towards the technology was the lowest concern, with a 2.62 score (representing a “high” concern). The results from both the organizational implementation readiness and implementation concerns assessments will be compared to the LCA groupings described in the following section.

Results from the LCA indicated that the optimal fit supported a three-class structure from the organization's self-reported technology use, with lowest BIC, adjusted AIC, and CAIC. The average posterior probability for each class was >.74, with class 1 = .932, class 2 = .866, and class 3 = .948 (Table 4). Total percent of overlap/cross-membership was only 1.9%.

**Figure 1:**
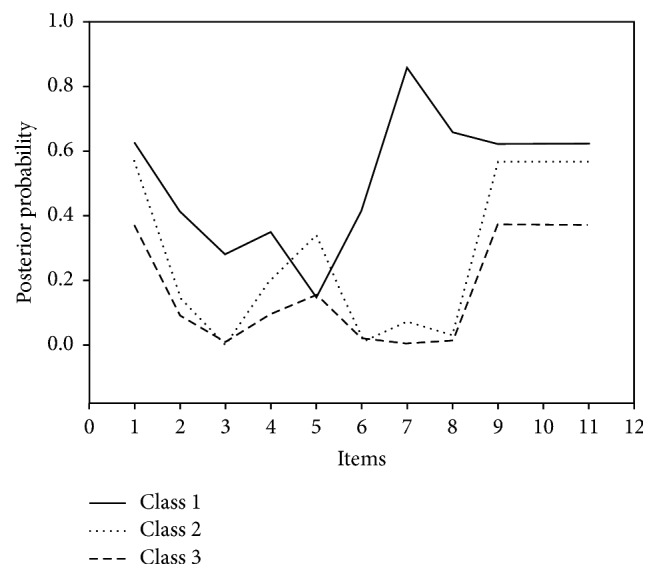
Latent Class Analysis (LCA) Estimated Probabilities. 1: computerized screening/assessments; 2: texting appointment reminders; 3: texting motivation messages; 4: organizational web portal; 5: video-based therapy; 6: mobile apps for use during treatment; 7: mobile apps for use after treatment; 8: secure chats for recovery supports; 9: telephone-based therapy; 10: telephone-based posttreatment recovery; 11: virtual worlds for treatment.

The three groupings based on the use rates of the 11 technologies were characterized as follows: (1) high overall technology use or “Innovators” (*n* = 27: 7.4%) (Class 1), (2) high for traditional technologies only (e.g., phone and video) or “Technology Traditionalist” (*n* = 101: 27.8%) (Class 2), and (3) low overall technology use or “Low Tech” (*n* = 235: 64.7%) (Class 3) (Figure 1). “Innovators” (Class 1) had high interest in all technologies except for video-based therapy. Class 2 or “Technology Traditionalists” showed a preference for the more established technologies such as web portals, video-based therapy, and phone-based technologies. Class 3 or “Low Tech” was lower than Classes 1 and 2 for nearly all technologies. A comparison of the different classes and organizational traits uncovered a few associations. The “Innovators” (Class 1) differed significantly from “Low Tech” (Class 3) by the readiness trait of “Our leaders are innovative” (*p* = .002). But, there was no significant difference between Technology Traditionalists and the other classes for “Our leaders are innovative.” The “Innovators” (Class 1) also differed from the “Technology Traditionalists” (Class 2) and “Low Tech” (Class 3) for the readiness traits of (a) “our clinicians and staff work well together” (staff cohesion) (*p* = .001) and (b) “Our departments work well together,” with both being significantly higher in the Innovator organizations.

## 4. Discussion

Internationally, alcohol results in 3.3 million deaths each year [30]. Opioid overdoses have become the leading cause of accidental death in the United States [31]. The prevailing paradigm of SUD treatment is through face-to-face therapy sessions that are sometimes provided in combination with SUD treatment pharmacotherapies. Telemedicine applications can potentially broaden access to SUD information, services, and support. Several telemedicine technologies have emerged to augment traditional treatment approaches, with many supported by research evidence [9, 32, 33]. Counselors understand and appreciate the need to connect with patients between appointments [34]. Similarly, patients want the safety net of support and community that digital technologies can provide.

In our study of 363 SUD organizations, the two telemedicine technologies that generated the most interest were computerized screenings/assessments and texting appointment reminders. Interestingly, both technologies represent opportunities to increase face-to-face clinical time with the patient. Computerized assessments reduce time needed to collect demographics and other background information, allowing counselors more time to discuss clinical issues. Texting appointment reminders has been found to reduce appointment no-shows [35]; higher show rates result in more clinical time with patients.

Organizations and their clinicians are also interested in increasing the clinical and social supports available to patients outside the face-to-face clinical encounters, as evidenced by the fact that two of the three largest gaps between interest in technology and reported use in the study were (a) mobile apps for posttreatment recovery at 46.56%, and (b) recovery support chats at 46.55%. The largest interest to use gap was with texting appointment reminders, at 55.18%. These gaps represent potential areas of telemedicine growth in American SUD treatment settings.

According to the LCA analysis, those in the “Innovator” organizations were more likely to use texting and mobile app technologies than those in the “Technology Traditionalist” and “Low Tech” organizations. This difference may be based on Innovators having the greater staff and departmental cooperation needed to implement these technologies. This could be because a staff person other than the clinician is often utilized to send the text or engage in the mobile app activities. Also, to gain access to the information generated by the mobile apps, clinicians typically need other staff to secure information from the mobile app server. In some cases, the electronic health record needs to be redesigned to integrate patient information from the mobile app into the traditional clinician workflows. Another observation was that “Low Tech” organizations were less likely than “Technology Traditionalists” and “Innovators” to use telephone-based recovery and therapy services. Of the technologies assessed, telephone services were the lowest tech services. Hence, Low Tech organizations lack a history of technology adoption that promotes embracing the more advanced technologies. In the LCA analysis, study participants' concerns with information security, regulation, and reimbursement did not create differences between the LCA use classes. Organizational history, leadership, and interdepartment as well as interpersonal cooperation created the differences in use between classes.

Several findings could be considered as contributions to the technology adoption science and studied in future research on technology. First, the technologies that were most frequently used supported existing traditional face-to-face clinical care practices. Second, organizational categories emerged based on the frequency of technology adoption. For example, “Innovators” and “Technology Traditionalists” had greater technology use than “Low Tech” organizations, and types of technologies adopted differed between the “Innovators” versus “Technology Traditionalists” categories. Lastly, the study confirmed the role of leadership and having a history of innovation in technology adoption. But, it also noted the importance of staff teamwork and interdepartmental cooperation in the adoption of certain technologies. Determining how to activate these roles should be part of future research on interventions in technology adoption.

## 5. Limitations

Several limitations exist. First, the data used in the analysis is based on self-report data. Hence, what is perceived as using a technology could differ from organization to organization. Second, organizations could vary in their interpretations of the definitions of the different technologies. For example, a web portal for one organization could be simply a web page, while for another, it could be a secure portion of a webpage that allows secure exchange of clinical information. Third, the response rates in three of the states were below 60%. This could affect the generalizability of the findings. In addition, this sample only represented eight of the 50 United States.

## 6. Conclusion

These data suggests that SUD treatment organizations in the United States are interested in greater use of telemedicine technology. Use of telemedicine in SUD treatment settings will probably begin with computerized assessments and texting appointment reminders, followed by the use of telephone, video, and mobile health applications. Organizations pursuing these goals will have demonstrated innovative tendencies in other organizational practices and have top leadership supporting the use of telemedicine. SUD treatment outcomes need to be improved and overdose deaths need to be decreased. Telemedicine could be a mode to achieve these desired goals or, at the least, provide new methods for delivering SUD treatment and recovery supports.

## Figures and Tables

**Table 1:** Survey participation rates.

State	Surveys completed	Surveys distributed	Return rate
Illinois	73	132	55.3%
Iowa	24	24	100%
Kentucky	10	10	100%
Massachusetts	73	130	56.2%
Ohio	58	65	89.2%
Oklahoma	71	74	95.9%
Oregon	25	59	42.4%
South Carolina	29	29	100%

Total	363	552	65.7%

**Table 2:** Technology interest and use.

Technology	% High interest	Relative rank	% Currently using	Relative rank	% Difference (interest – use)	Relative rank
Computerized screening/assessments	69.97%	1	44.63%	1	25.34%	9
Texting appt. reminders	68.40%	2	13.22%	6	55.18%	1
Web portal for patients	58.40%	3	14.60%	5	43.80%	4
Mobile apps for posttreatment recovery	55.65%	4	9.09%	7	46.56%	2
Video-based therapy	54.82%	5	20.39%	4	34.43%	8
Telephone-based recovery support	53.99%	6	29.48%	2	24.51%	10
Recovery support chats	53.44%	7	6.89%	8	46.55%	3
Telephone-based therapy	49.04%	8	28.37%	3	20.67%	11
Texting motivational messages	45.18%	9	2.48%	10	42.70%	5
Mobile apps for treatment	40.77%	10	4.96%	9	35.81%	6
Virtual worlds	35.54%	11	0.55%	11	34.99%	7

**Table 3:** Technology implementation concerns inventory results.

Item	Average	Organizational concern
Informational security	3.90	Very High
Reimbursement policy towards the technology	3.78	Very High
Regulation barriers	3.53	Very High
The technology's impact on workflow	3.25	High
Counselor attitudes toward the technology	2.71	High
Patient attitudes toward the technology	2.62	High

**Table 4:** Latent class analysis of technology use: goodness-of-fit statistics and likelihood ratio tests.

Class	Entropy	BIC	AIC	Adj AIC	CAIC
1	-	3038.434	2995.596	3003.536	3049.434
2	0.693	2928.513	2838.942	2855.544	2951.513
**3**	**0.811**	**2908.36**	**2772.061**	**2792.326**	**2943.366**
4	0.721	2952.646	2769.61	2803.536	2999.646

Class 1  *N* = 27 (7.4%), Class 2  *N* = 101 (27.8%), and Class 3  *N* = 235 (64.7%).
